# Automatic annotation to train ROI detection algorithm for premature infant respiration monitoring in NICU

**DOI:** 10.1007/s11517-026-03596-y

**Published:** 2026-06-24

**Authors:** Ádám Nagy, Péter Földesy, Imre Jánoki, Máté Siket, Zita Róka, Ákos Zarándy

**Affiliations:** 1 HUN-REN Institute for Computer Science and Control, 13-17. Kende street, H-1111 Budapest, Hungary; 2 Faculty of Information and Bionics, Pázmány Péter Catholic University, 50/A Práter street, H-1083 Budapest, Hungary

**Keywords:** Computer Vision, Automatic annotation, Image processing, NICU

## Abstract

**Abstract:**

Visual monitoring of vital parameters in premature infants has become an intensively studied area in recent years. Among these parameters, respiration rate (RR) is one of the most critical vital signs, making non-contact measurement of respiration a key research focus. Many published algorithms achieve improved performance when an appropriate region of interest (ROI) is detected prior to RR estimation. Typically, such ROIs are generated using data-driven segmentation methods. However, modern deep learning–based ROI detection algorithms require thousands of annotated samples for training, and manual data collection and annotation are time-consuming and labor-intensive. In this work, we propose a motion–periodicity–based method to automatically generate respiration-related region masks that capture the abdominal or chest area of neonates. The predicted masks were validated against independent expert annotations, achieving high localization consistency on the torso ($S_{geo}=0.987$) and significant overlap with clinical ground truth (mean IoU=0.580 and Dice=0.735). We further show that these automatically produced labels can be directly used to train common segmentation architectures, eliminating the need for manual annotation and enabling the creation of large, high-quality datasets for neonatal respiration analysis. Our findings demonstrate that automatic dataset generation is both feasible and effective for training deep learning–based ROI detectors in this domain.

**Graphical abstract:**

**Figure:**
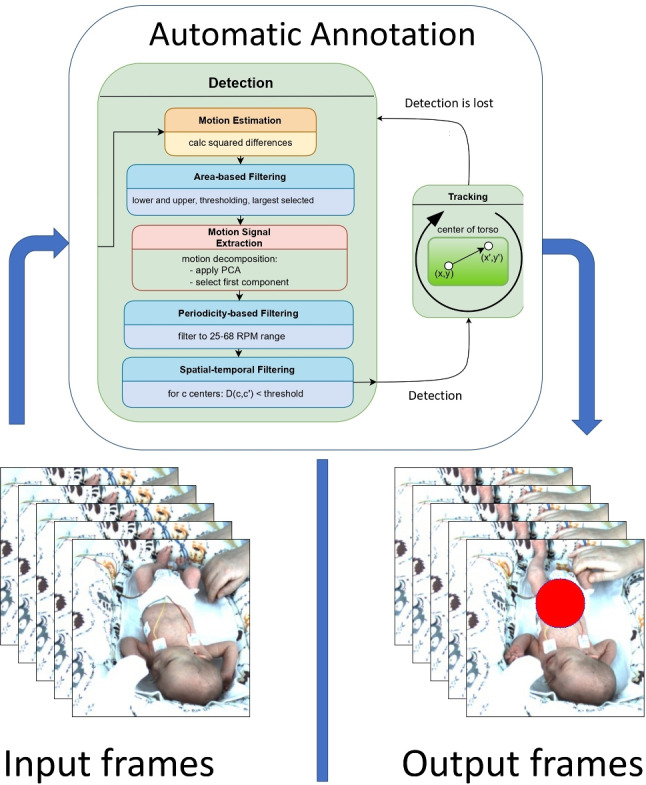

## Introduction

Non-contact monitoring of neonatal respiration rate has emerged as an important and extensively investigated area of research [1–6]. Accurate and robust respiration rate (RR) estimation is an important prerequisite for downstream clinical analyses, including the assessment of abnormal breathing patterns. Obstructive sleep apnea (OSA) is a frequent condition in infants and may indicate a variety of underlying disorders. Nasal occlusion can induce a switch to mouth breathing in approximately 40% of newborns, which in turn predisposes them to OSA [7]. Moreover, respiratory distress occurs in around 6.4% of neonates [8]. Continuous respiratory monitoring is particularly important for premature infants, as it provides essential longitudinal information on respiratory dynamics that may support future clinical assessments.

Several studies have reported associations between gastroesophageal reflux and OSA [9]. Another clinically relevant respiratory phenomenon is apnea – the transient cessation of breathing [10]. These conditions illustrate the broader clinical context in which reliable respiration monitoring is required, although their detection is beyond the scope of the present study. Beyond pathology detection, respiration monitoring also provides valuable information on sleep quantity and quality, which are key factors in healthy neonatal development [11].

Several studies have reported that accurate region-of-interest (ROI) detection can significantly enhance the performance of respiration rate (RR) estimation algorithms [12–17]. A comprehensive review of RR estimation methods is provided in [1], which also summarizes the ROI detection strategies commonly employed. These approaches are increasingly data-driven, and many are based on convolutional neural networks (CNNs).

However, deep learning–based ROI detection methods typically require large numbers of annotated images. Data collection and manual annotation are time-consuming and resource-intensive processes, often demanding specialized software and significant human effort. In our previous work [6], we introduced a U-Net–based neural network designed to detect the torso as the ROI for RR estimation. One of the main challenges we faced was generating a sufficiently large, high-quality annotated dataset to train this network – highlighting the need for an automated annotation strategy.

In the present study, we build upon our earlier system capable of non-contact monitoring of respiration and pulse in preterm infants using an overhead camera mounted on an incubator. The novelty of this work lies in the introduction of a new algorithm that enables automatic annotation of the data required to train the non-contact respiration monitoring ROI detector.

In this paper, we propose the Automatic Labeling Algorithm (ALA) for fully automated annotation. This motion-periodicity–based method is designed to identify the regions of the torso that exhibit active movement during respiration. These areas, referred to as the Respiration Region of Interest (R-ROI), typically correspond to the lower abdomen or back, slightly above and partially overlapping with the diaper region.

The ALA operates solely on the spatial extent and periodicity of motion observed in the image sequence, requiring no manual input or human annotation. This approach offers several advantages. First, it eliminates the labor-intensive process of manual data labeling. Moreover, it produces an automatically annotated dataset that can, in some cases, surpass human annotations when used to train the segmentation component of the respiration extraction pipeline.

The key reason is that human annotators typically mark static regions (e.g., the abdomen or back) based on still frames, without perceiving the spatio-temporal dynamics of breathing. In contrast, ALA analyzes extended video segments to derive labels that inherently capture motion periodicity, ultimately improving the accuracy of downstream respiration rate estimation, which is the sole performance outcome evaluated in this study. As demonstrated in the RR evaluation results, ROI masks generated from ALA-derived annotations enabled stable and physiologically meaningful respiration rate estimation across independent subjects. Although subject-wise cross-validation (e.g., LOSO) was not feasible due to limited availability of synchronized respiratory ground-truth signals, per-infant evaluation with uncertainty estimation was performed to quantify inter-subject variability and robustness.

The rationale for not using the ALA directly as a ROI detector during respiration rate computation is detailed in the Discussion section.

In this work, we make several key contributions. First, we introduce the Automatic Labeling Algorithm (ALA), a motion-periodicity–based method that automatically generates respiration-related ROI masks (R-ROIs) from neonate video recordings, completely eliminating the need for manual annotation. Second, we demonstrate that these automatically generated labels can be used to train deep learning models, including U-Net and U-Net++, which then achieve higher accuracy in detecting R-ROIs and estimating respiration rates than networks trained on manually annotated datasets. Finally, we validate our approach on real NICU recordings, showing a 96% agreement between automatically generated masks and manually annotated regions. These contributions are presented and evaluated in the following sections.

While accurate RR estimation is a prerequisite for many downstream clinical analyses, the present work focuses exclusively on automatizing the annotation process for respiration-related regions and does not address the detection or prediction of clinically significant respiratory events. No claims are made regarding clinical event detection, diagnosis, or outcome prediction, and these remain important topics for future work.

## Dataset

**Fig. 1:**
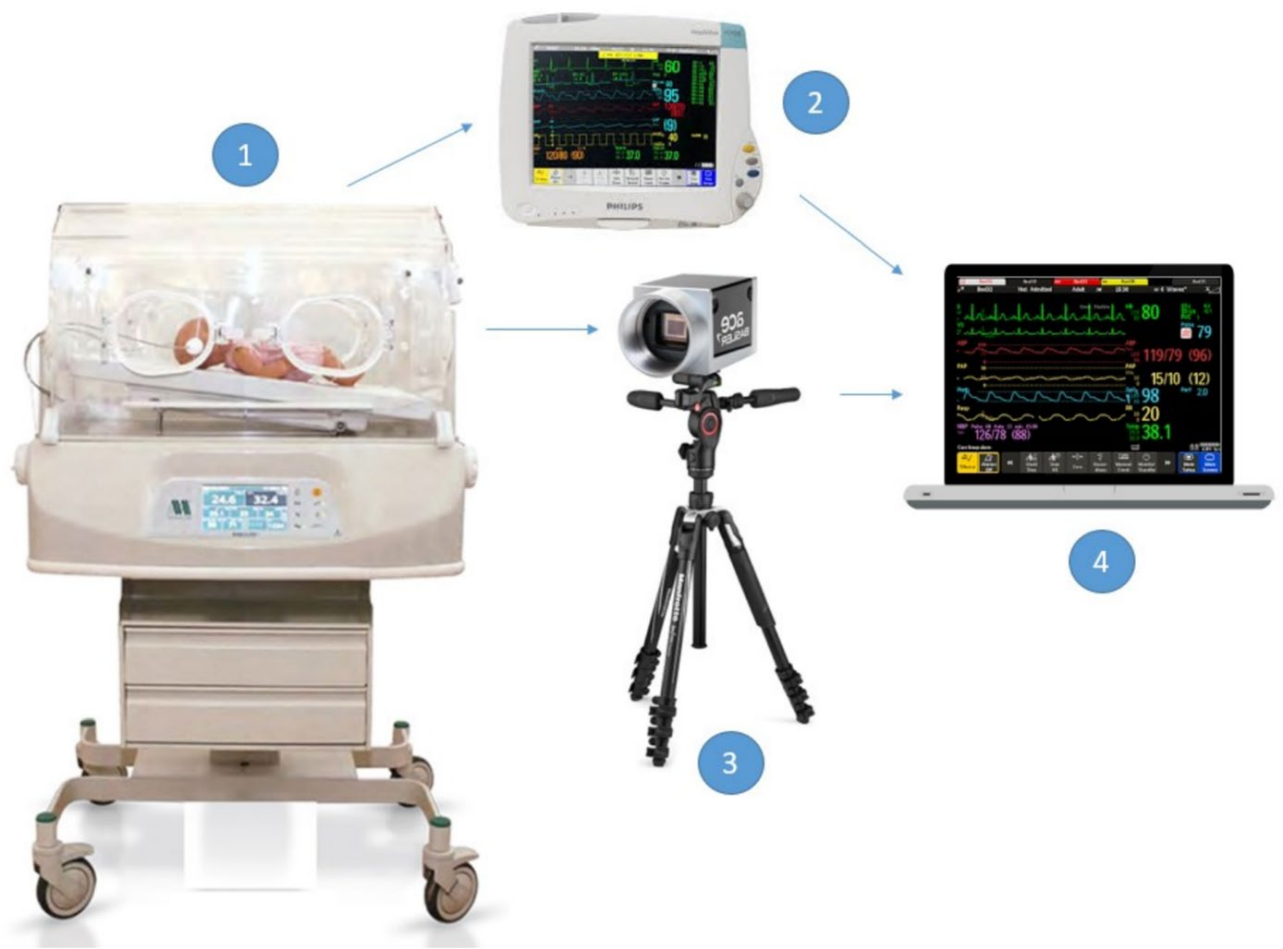
In the experimental setup the neonatal infant in NICU (1) was monitored with a Basler acA2040-55uc RGB camera (3). At the same time, physiological signals were received from the Philips IntelliVue MP20/MP50 monitors (2). The wave and rate data from the monitors and the video images from the camera were saved in sync to a laptop (4)

Image data for automatic annotation were acquired using an industrial camera mounted above neonatal incubators. Physiological signals were recorded simultaneously and synchronized with the video data. The complete dataset was collected in the Neonatal Intensive Care Unit (NICU) of the 1st Department of Pediatrics and the 2nd Department of Obstetrics and Gynecology at Semmelweis University, Budapest, Hungary. The experimental setup used for data collection is illustrated in Fig. 1. Further details about the data acquisition system are provided in [6].

The camera captured raw images at a resolution of 500 *×* 500 pixels and a frame rate of 20 frames per second. To enhance generalizability, recordings were performed from multiple viewing angles and included several different infants. The distance between the Basler acA2040-55uc camera and the subject varied between approximately 80 cm and 1.5 m. Participant demographics–including age, weight, sex, respiratory support status, and medication use–are summarized in Table 1. In addition to the 10-infant cohort summarized in Table 1, we evaluated the trained model on one additional independent infant recorded under the same acquisition protocol but not included in the training/validation/test split. This subject was not part of the dataset used for model development and is therefore considered an external evaluation case.

**Table 1:** Demographic properties of the population of participants

Subject	1	2	3	4	5	6	7	8	9	10
Recording time (hours)	96.7	5.5	39.4	27.4	51	105.5	50.1	36.4	56	38.2
Gender	F	M	M	F	F	M	F	F	F	M
Gestational age (weeks)	32	32+3	31+4	35+4	39	32	33	38+6	24+2	33+4
Birth weight (g)	2020	1840	1850	1870	3150	2120	2080	2840	760	2100
Postnatal age (days)	4	4	10	8	4	7	2	7	11	1
Actual weight (g)	1900	1850	1680	1820	2905	2040	1960	3150	750	-
Length (cm)	46	44	-	45	57	45	44	48	46	45
Head circumference (cm)	32	29.5	-	32	34	30	32	33	22	-
Respiratory support	no	no	no	no	no	no	no	yes	yes	no
Any drugs	no	no	no	no	yes	no	no	yes	yes	yes
Fitzpatrick scale	2	3	2	2	2	2	2	2	2	2

## Related works

Recent years have seen substantial progress in non-contact respiration rate (RR) monitoring, particularly for infants in open incubators [18–21]. A broad overview of these video-based approaches is provided in [1]. In adults, however, most studies focus on monitoring chest motion within a manually defined region of interest (ROI) [22, 23], reflecting a difference in both imaging setup and motion characteristics between infant and adult populations.

Maurya et al. offered an extensive review of respiration sensing technologies, including RR estimation algorithms and ROI detection techniques [1]. Their survey highlighted a clear shift toward deep learning–based segmentation, as also supported by comparative evaluations in [17], which identified CNN-based methods as the most reliable. Likewise, Jorge et al. emphasized that robust CNN-driven subject segmentation substantially enhances the performance of non-contact vital sign monitoring systems [12], while Nagy et al. demonstrated measurable gains from accurate ROI detection [6]. Collectively, these studies establish CNN-based ROI segmentation as a cornerstone of modern, non-contact physiological signal monitoring.

Complementary research has explored motion- and periodicity-based detection methods for identifying relevant dynamic areas [24–27]. Parallel efforts have emerged around automatic annotation, aiming to generate labeled datasets without manual effort. Although many such methods are not always camera-based, their conceptual similarities are noteworthy. For instance, Du-Ming Tsai et al. and Yuwei Xia et al. applied CycleGAN-based frameworks to create automatically annotated datasets for defect segmentation on industrial surfaces [28, 29]. Several studies–such as [30–34]–have employed automatically annotated or synthetic datasets to train neural networks for medical semantic segmentation tasks. Other works have focused on generating synthetic medical datasets [35–38]. In [39], for example, the authors generated labeled healthy and abnormal electrocardiogram (ECG) signals for arrhythmia detection.

Further developments have integrated CNNs with optical flow estimation to enhance motion-based segmentation. It is presented for example, in [40], where the authors combined convolutional neural networks with optical flow analysis to segment moving objects in video sequences. Similarly, [41] investigates the performance of deep learning–based optical flow estimation across various tasks, including unsupervised and semi-supervised learning scenarios.

It is evident that automatic annotation or synthetic dataset generation is a widely adopted strategy for researchers who require large volumes of labeled data. However, to the best of our knowledge, its application in respiration rate (RR) monitoring remains novel. Furthermore, the proposed ALA algorithm is unique in that it bridges the gap between dynamic label generation and training a static network, ultimately enabling efficient analysis of dynamic scenes using an intra-frame approach. It is important to note that the ROI detectors presented in [12–14] demonstrate excellent performance, but these methods rely on large, manually annotated datasets or training. The primary contribution of our work is the introduction of a method to automatically generate annotated datasets for RR-ROI detection without any human input, thereby eliminating inter-observer variability while maintaining robust performance.

## Methods

### The Automatic Labeling Algorithm (ALA)

We propose the Automatic Labeling Algorithm (ALA), a motion- and periodicity-based method to generate an automatically annotated dataset. It can be divided into two primary components, which will be described in detail below:

- R-ROI detection
- R-ROI tracking

**Fig. 2:**
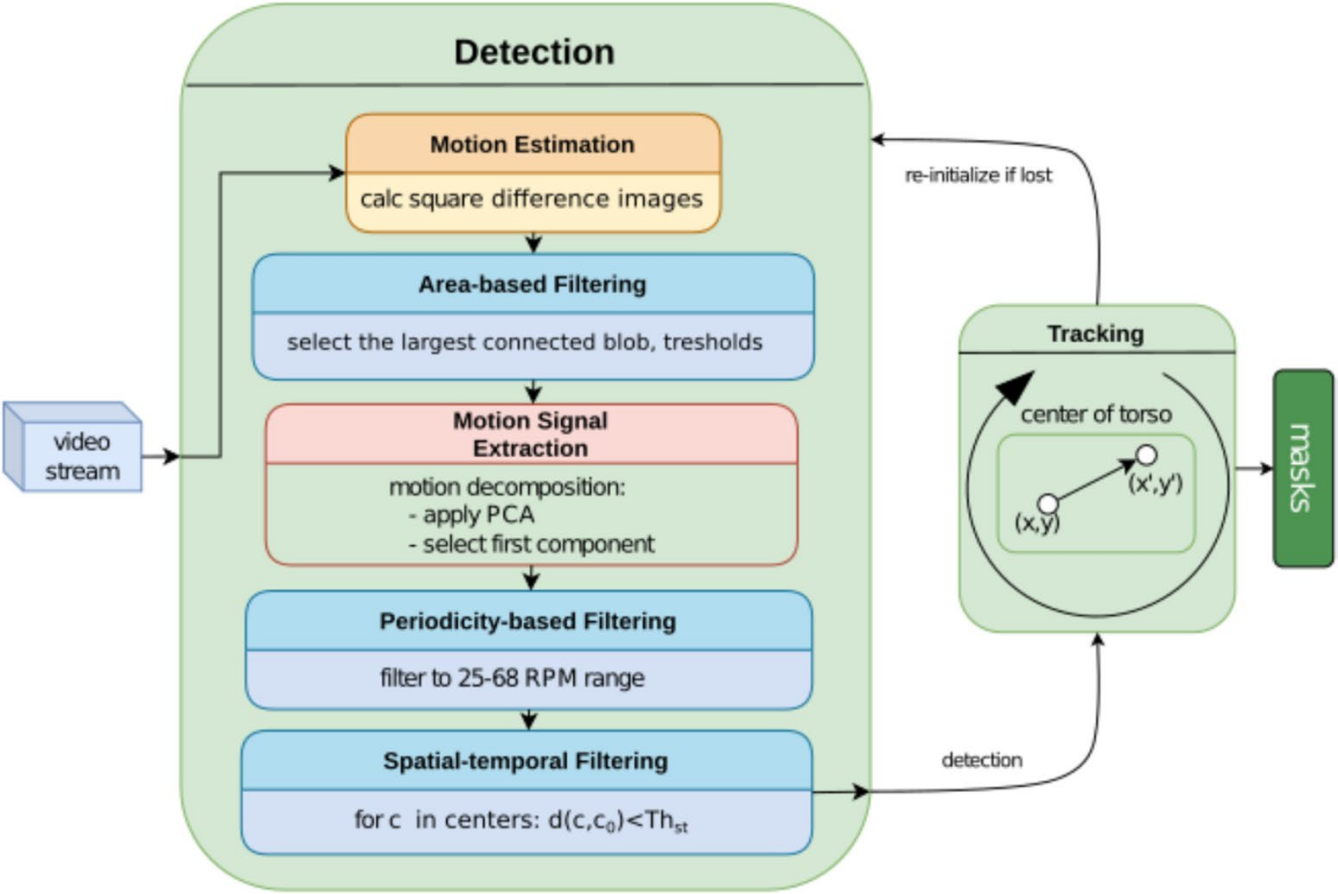
The automatic annotation algorithm (ALA). The input is a video stream, a series of consecutive frames, and the output is a series of binary images where the R-ROI of the infants is masked. The connection of the detection and the tracking is shown in the figure. The steps for "R-ROI Detection" and tracking are detailed below

R-ROI detection is performed by analyzing the motion patterns and the periodicity of the observed movement. Rather than detecting the R-ROI in every frame, the algorithm identifies it once with high confidence and subsequently tracks it for as long as it remains visible. If tracking is lost due to occlusion or external interference (i.e. a clinician’s hands overlay the R-ROI), the detection step is re-initialized. During successful tracking, automatic annotation and R-ROI labeling are performed continuously. An overview of the complete workflow is illustrated in Fig. 2.

### Detection

A key assumption of our method is that, when the camera observes a neonate in the incubator (as described in the experimental setup below Fig. 1) and the infant is breathing normally, the position of the R-ROI can be determined based on the periodicity of the respiratory motion.

An overview of the periodicity-based R-ROI detection is shown in Fig. 2, which summarizes the steps of this process too, under “Detection”:

1. Motion Estimation
2. Area-based Filtering
3. Motion Signal Extraction
4. Periodicity-based Filtering
5. Spatio-temporal Filtering

An R-ROI detection is accepted only if all filtering steps yield positive results; otherwise, the current period is discarded and no annotation is generated.

#### Motion estimation

Several algorithms exist for quantifying motion in video frames, such as optical flow, Deep Flow [42], or block-matching methods [43], which perform well in estimating both the magnitude and direction of movement. However, for this application, estimating motion direction is not required. Instead, we employed morphological and frequency analysis of difference images for motion estimation, as this approach directly yields a single-channel motion intensity map that is rotation-invariant.

**Fig. 3:**
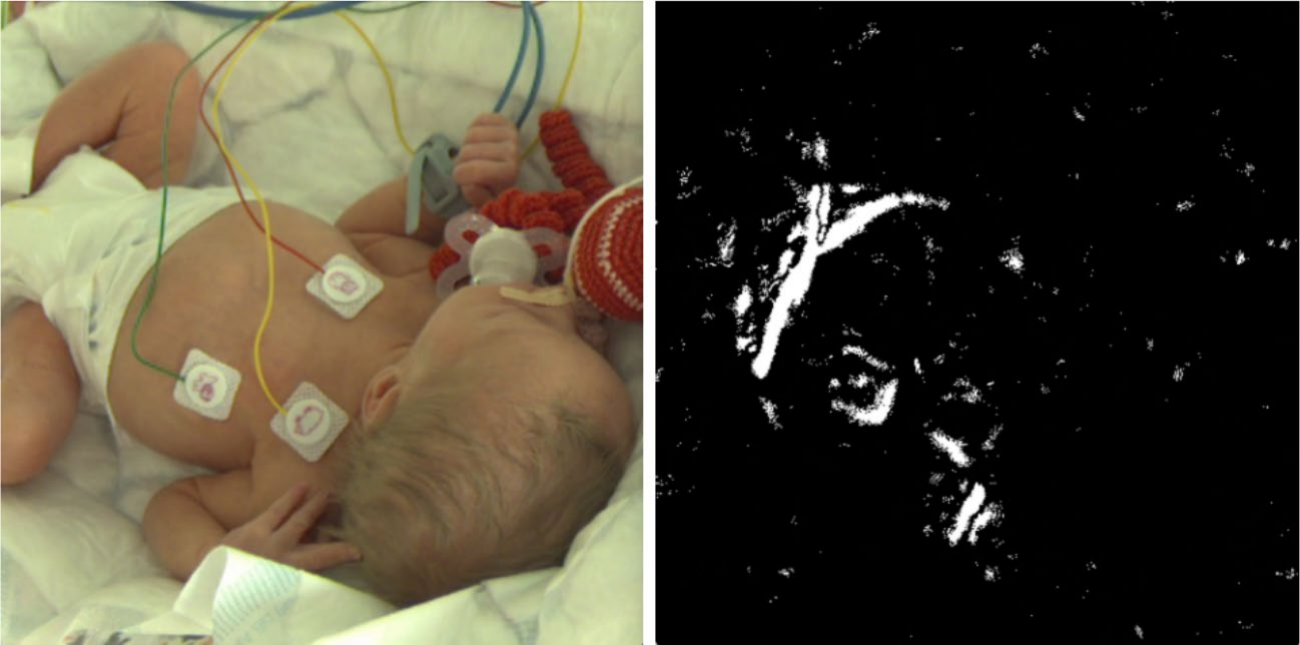
Motion-estimation: The current input image can be seen on the left side, while the squared difference image computed according to (1) between the current and previous images is shown on the right side

To detect the R-ROI, we computed the squared difference between consecutive frames (${\textbf {D}}$) to quantify motion at each pixel, as defined in Eq. (1). Squaring the differences amplifies stronger motions while attenuating weaker ones, thereby emphasizing the most intense movements (Fig. 3). Using these difference images, we applied a motion area–based detection method to localize the R-ROI.

1 $$\begin{aligned} \textbf{D}(x,y,n) = (\textbf{I}(x,y,n) - \textbf{I}(x,y,n-1))^2, \end{aligned}$$

where $\textbf{I}(x,y,n)$ means the grayscale pixel intensity in coordinates (x,y) at the $n^{th}$ frame, and $\textbf{D}(x,y,n)$ is the (*x*, *y*) pixel value of the difference image at the $n^{th}$ frame.

#### Area-based filtering

If the neonate’s respiration is normal and calm, with no additional motion present, a threshold can be applied to the difference image to generate a binary mask. Following thresholding, morphological dilation is performed, after which the algorithm identifies the largest blob in the frame, which is considered the R-ROI.

For detecting the largest connected region, we employed the Spaghetti algorithm [44], which combines the Binary Block Decomposition Tree mask with a state prediction paradigm to address the connected component labeling problem. The method constructs reduced decision trees and merges them into a directed rooted acyclic graph to assign labels to non-zero pixels in the input binary image based on their connectivity.

As described above, the algorithm ultimately selects the largest connected component in the frame as the R-ROI.

2 $$\begin{aligned} LR(x,y)= {\left\{ \begin{array}{ll} 1, & \text {if } l(x,y)=L \\ 0, & \text {otherwise} \end{array}\right. } \end{aligned}$$

where *l*(*x*, *y*) is the label that is generated by the Spaghetti algorithm for (*x*, *y*) place from the current square difference and *L* is the label that can be associated to most of the pixels (which means it is the label of the largest region).

**Fig. 4:**
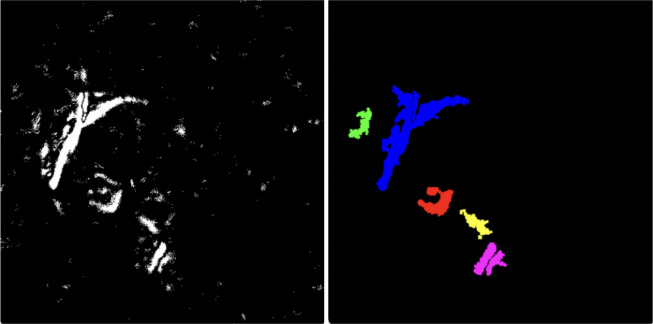
Area-based Filtering: The difference image computed in the previous step is shown on the left side, while the connected regions found within it is on the right side. Among these, we select the largest connected region corresponding to the "L" label, which in this case is represented by the blue region

In this way, the largest connected region is selected. After this, a lower and an upper threshold for the area of the regions is applied and those frames are omitted which contain an unrealistically small or large detected region. In other words, $Th_{lower}< \sum _{x,y=1,1}^{M,N} LR(x,y)< Th_{upper}$ must be fulfilled (Fig. 4).

#### Motion signal extraction

Area-based filtering performs well when only respiratory movements are present in the video. However, when the infant is active, limb movements can produce difference patterns that are larger or comparable in size to the respiratory motion. To address this, we additionally apply time-domain filtering based on periodicity.

For this purpose, a one-dimensional motion signal is generated using principal component analysis (PCA) [45]. A sliding window containing 300 frames, including the current frame, is employed. Each frame is first flattened into a vector, and these 300-length vectors are then stacked as rows of a large matrix:

3 $$\begin{aligned} \textbf{X} \in \mathbb {R}^{a \times b} \end{aligned}$$

where *a=300* and *b* is the number of pixels on the individual frames and *X* contains the values of the pixels of flattened frames in the sliding window mentioned above.

The PCA transforms our data to a new coordinate system in a way that the data projection with the greatest variance comes to lie on the first coordinate, the projection with the second greatest variance on the second coordinate, and so on. After, we use the PCA result that is related to the first component, which provides a 1-dimensional motion signal ($\textbf{s}_M$) containing 300 data points.

#### Periodicity-based filtering

Detecting only the moving areas is insufficient to reliably distinguish respiratory motion from other movements, such as limb motion, that may have comparable spatial extent. Another distinguishing feature of respiratory motion is its characteristic frequency. Newborns typically breathe within the 25–68 RPM range [46]. The motion signal extracted from the sliding window described above is filtered based on whether its dominant frequency falls within this respiratory range. For frequency estimation, the fast Fourier transform (FFT) of the motion signal is computed, and the frequency corresponding to the largest spectral peak is selected.

4 $$\begin{aligned} RPM = \textbf{x}[\max \limits _{f} \left\{ FFT(\textbf{s}_M)\right\} ], \end{aligned}$$

where $\textbf{s}_M$ is the 1-dimensional motion signal extraction calculated from the previously mentioned sliding window, whereas $\textbf{x}$ is a vector that contains the frequency bins of the FFT spectrum.

If the highest peak is not in the said frequency range, there is no detection and the particular frame is discarded, because we cannot guarantee that the selected moving region belongs to a belly or a back with respiratory movement. In other words, *RPM* must be in the range of $[Freq_{lower},Freq_{upper}]$ or there is no detection. The choice of the frequency limits of this range originates in the physiologically relevant range (40–120 BPM).

#### Spatio-temporal filtering

After periodicity-based filtering, spatio-temporal filtering is applied to limit the maximum displacement of the selected region. During calm periods, it can be assumed that the geometric center of the largest region does not shift abruptly from its initial position. To enforce this, a secondary time window is applied, containing the centers of the largest regions (if detectable) across consecutive frames. If none of the centers within this window deviate from the initial center by more than a predefined threshold, the current difference image is accepted. When the largest connected region satisfies this criterion and is considered an R-ROI–related pattern, the tracked center is updated to the geometric center of this region, and tracking continues. In other words, Equation 5 must be satisfied for a valid detection.

5 $$\begin{aligned} \forall c_{i} \in \textbf{c}, d(c_{i},c_{0}) < Th_{st} \end{aligned}$$

where *i<n*, *d* is the “Euclidean distance”, $\textbf{c}$ is a vector that contains n=300 peaces of 2D center points ($c_{i} \in \mathbb {R}^{2}$).

### R-ROI tracking

After detection, sparse optical flow [47, 48] is used to track the R-ROI. In our approach, the algorithm follows a single point corresponding to the geometric center of the largest region in the accepted difference image. During tracking, a mask is drawn around the tracked center points, marking the portion of the R-ROI where respiratory movements are observable.

A major advantage of optical flow is its relative robustness to illumination changes and shadows, coupled with low computational cost. It can track the point even when the neonate exhibits significant movement. However, tracking may be lost if the neonate’s arm or another object crosses the path of the tracked point, or if a caregiver intervenes in the frame. In such cases, the algorithm reverts to the detection phase, as illustrated in Fig. 2.

**Fig. 5:**
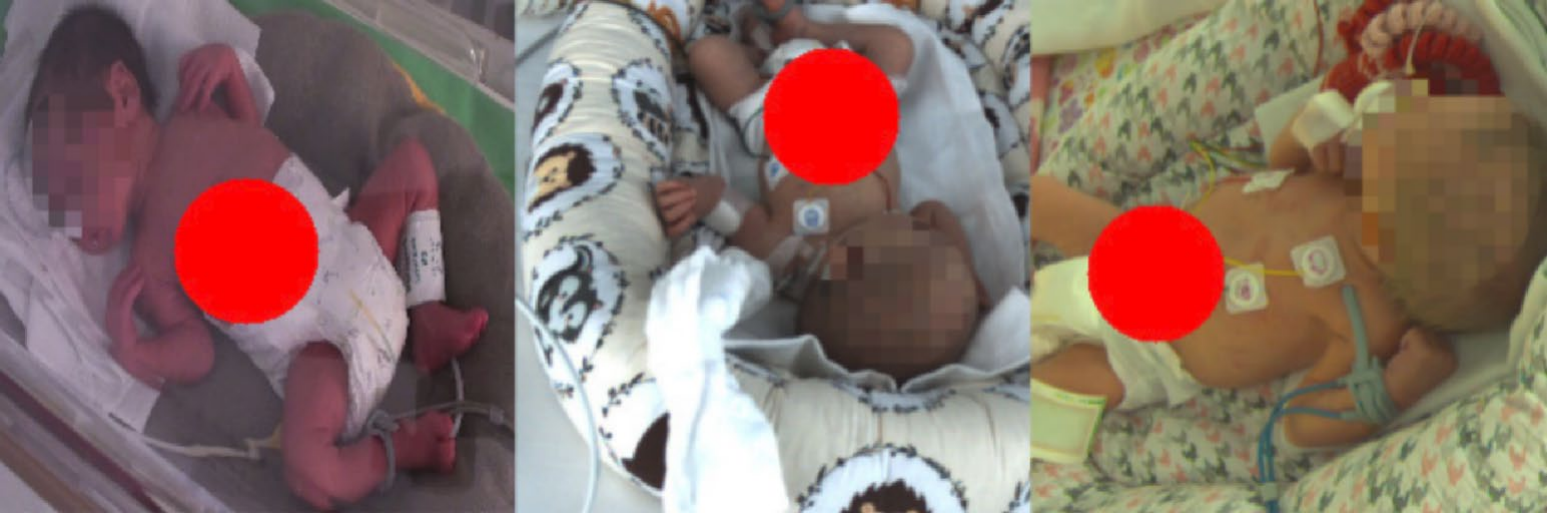
The algorithm draws mask around the tracked center points. For the automatically labelled dataset, these masked images are saved in every 10 seconds.=

Initially, all original camera frames and the corresponding automatically annotated binary masks were considered as input-label pairs. However, consecutive frames are highly similar due to the short time interval between them. To reduce redundancy, we selected one image pair every 10 seconds to construct the automatically labeled dataset (see Fig. 5).

### Training

In this work, motion-based R-ROI detection and tracking were employed to generate an automatically annotated dataset, which was subsequently used to train a more robust deep learning–based model. We trained a U-Net architecture [49], a proven and effective deep learning structure for semantic segmentation tasks. U-Net is straightforward to implement, computationally efficient, and requires relatively few labeled images–in our experience, a few thousand.

**Fig. 6:**
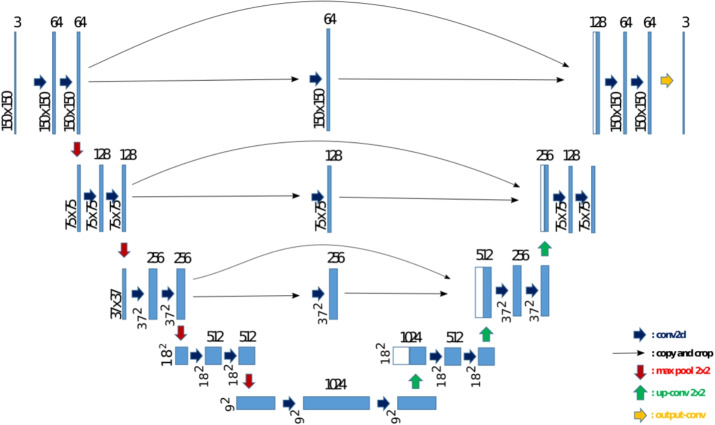
The modified *U-Net++* architecture working on different spatial resolution than the traditional ones. The input image is of size 150x150 pixels. Note, that we used bigger kernels of size 5x5 instead of the traditional ones (3x3). The traditional U-Net architectures have some limitations. To address these limitations, U-Net++ is proposed for the purpose: (1) reducing the unknown network depth by efficiently combining U-Net models with different depths that partially share an encoder and learn together, simultaneously applying deep supervision; (2) re-designing the skip connections to aggregate feature maps of varying semantic scales in the decoder subnetworks, resulting in an extremely flexible feature fusion scheme; and (3) devising an intersection scheme to accelerate the inference speed of U-Net++ architecture

We used a variant of U-Net++ [50], which outperformed the traditional U-Net in our experiments. In addition, the original architectures employ kernels with a limited receptive field, whereas a larger field of view is desirable at the lowest resolution, since the positions of limbs and the head can provide valuable context when localizing the R-ROI. Therefore, 5*×*5 convolutional kernels were used instead of the traditional 3*×*3 kernels. The modified architecture is illustrated in Fig. 6. Our U-Net variant takes 150*×*150 RGB images as input and outputs binary masks indicating the infant’s R-ROI.

The generated dataset comprised 6,000 images extracted from recordings of 10 different infants. Each recording spanned approximately 24 hours, with around 600 images annotated by the algorithm per recording based on a predefined parameter. The dataset was partitioned as follows:

- Training set: 4,200 images from 7 infants
- Validation set: 600 images from 1 independent infant
- (Holdout) Test set: 1,200 images from 2 independent infants

In the training we used mean squared error (MSE) as the loss function and an Adam optimizer was used to train the network. We applied “online augmentation” and early stopping with the validation set as well. We also used "Comet-ML" (Comet.ml, NY, USA) for the visualization of loss, epoch loss, and for hyperparameter tuning.

### Respiration calculation

**Fig. 7:**
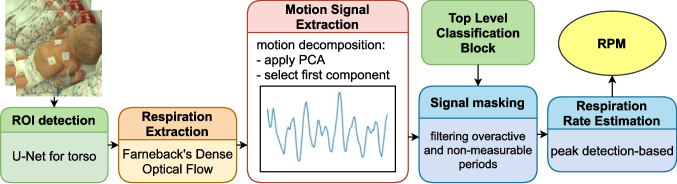
Overview of the Respiration-rate-estimator algorithm published in [6]. This solution applies a U-Net for ROI detection and generates RR from a stack of consecutive images

In our article [6], we introduced a respiration monitoring algorithm that employs a traditional U-Net as an ROI detector, trained using manually annotated R-ROI images. The algorithm incorporates a sliding window approach, in which a dense optical flow [51] extractor and a peak detection–based module are used to estimate the respiration rate. A flowchart of the algorithm is shown in Fig. 7. An implementation of the algorithm is available at the following GitHub repository: [52].

### Top level classifier

In our article [6], we also introduced a module called the TopLevelClassifier, designed to determine the infant’s current care status and quantify activity. This module not only measures the infant’s activity but also detects whether the infant is present in the incubator, and whether any care interventions or other moving objects, such as a caregiver’s hand, appear in the frame. On the dataset introduced in [6], this top-level classification achieved 97.9% sensitivity and 97.5% specificity.

By running the TopLevelClassifier on the input video prior to, or in parallel with, the ALA algorithm, we obtain additional information about whether the infant is in the incubator or if the measurement is being disturbed by external movements, such as those of caregivers.

### Efficiency

#### Runtime

To assess the computational requirements of the proposed approach, we measured the processing time of the full pipeline, including ROI detection and respiration-rate estimation, on a standard workstation equipped with an AMD EPYC @ 2.25 GHz CPU and 12 GB of RAM. The proposed U-Net++-based ROI detector achieved an average per-frame processing time of 0.0645 ± 0.02 s, corresponding to  15–16 frames per second.

#### Computational complexity

The U-Net++ model used for ROI detection consists of approximately 7.8 million parameters and requires 28 MB of memory, which is small enough for real-time inference on CPU-based systems.

## Results

To facilitate understanding and improve readability, we define the datasets and models used in this study. The datasets employed during the experiments are as follows:

- $D_{man}$: Dataset with manual labeling performed by three human annotators using the labelme annotation tool [53]. The mean Intersection over Union (mIoU) computed on the overlapping subset annotated by multiple raters was *0.86 ± 0.006*.
- $D_{ala}$: Dataset with automatic labeling generated by the ALA algorithm.

The following models were trained and evaluated:

- A traditional U-Net trained on $D_{man}$ ($U{{\text {-}}}Net_0$)
- A traditional U-Net trained on $D_{ala}$ ($U{{\text {-}}}Net_1$)
- A U-Net++ [50] trained on $D_{ala}$ (*U-Net++*)

It is important to note that, unlike typical segmentation tasks, our primary objective is not pixel-level accuracy but rather respiration rate (RR) measurement accuracy, although segmentation performance is also assessed in this section. RR measurement accuracy was quantified using the mean absolute error (MAE) and root mean square error (RMSE) metrics.

For ground truth, we used respiratory waveforms derived from impedance variations measured via ECG electrodes attached to the infants. Respiration rates computed from these waveforms, obtained from IntelliVue MP20/MP50 patient monitors, served as the primary ground truth.

Evaluation was conducted on two levels: first, the segmentation performance was assessed, and second, the RR measurement accuracy was analyzed.

### Segmentation performance analyses

**Fig. 8:**
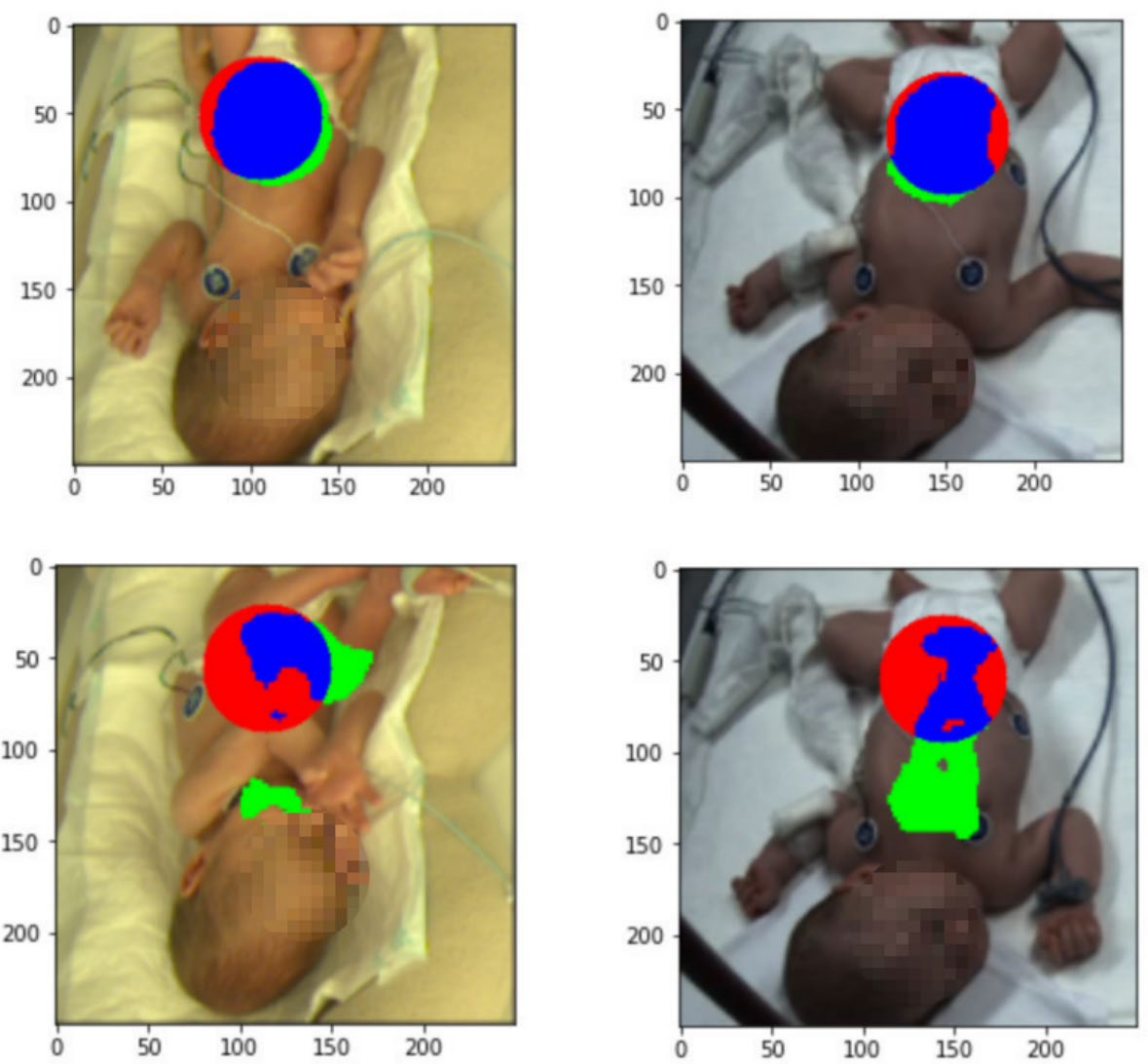
Results of the ROI detector where the detection was performed by a $U{{\text {-}}}Net_1$ trained on $D_{ala}$. The output of ($U{{\text {-}}}Net_1$) is green+blue, while the expected mask (generated by ALA) is red+blue. (Blue is their intersection.) In the first row, you can see some good examples where more than 50% of the detection (#blue pixels/#red+blue pixels) overlap with the expected mask (96% of the images), while the second row shows some bad examples where the overlap is less than 50%

We defined a morphological similarity measure to quantify how closely a U-Net–generated mask resembles an ALA-generated mask:

6 $$\begin{aligned} S_{geo}(M_U, M_A) = \frac{\text {number of pixels in } (M_U \cap M_A)}{\text {number of pixels in } M_A}, \end{aligned}$$

where $M_U$ denotes the binary mask generated by $U{{\text {-}}}Net_1$, and $M_A$ is the binary mask generated by ALA. $S_{geo}$ represents the ratio of the overlapping pixels (blue) to the total pixels in the ALA mask (red + blue) as illustrated in Fig. 8. The justification for using $S_{geo}$ in this context is to demonstrate that the predicted ROIs are consistently located within the physiologically relevant torso region, as manually defined by experts. It is important to clarify that this measure does not penalize the network for not covering the entire torso; since the models were specifically trained to identify localized respiratory areas rather than the whole body, the key requirement is that the predicted mask resides within the torso boundaries. This high degree of containment ensures that the ROI is positioned where respiratory motion is physically present and measurable, thereby fulfilling the functional goal of the segmentation. For the independent validation, two standard overlap metrics were employed to quantify the agreement between the predicted mask ($M_U$) and the expert-annotated manual mask ($M_{exp}$): the Intersection over Union (*IoU*) and the Dice Similarity Coefficient (*DSC*). While *IoU* provides a strict measure of overlap, *DSC* is often used in medical imaging to represent the harmonic mean of precision and sensitivity. These metrics are defined as:

7 $$\begin{aligned} IoU(M_U, M_{exp}) = \frac{|M_U \cap M_{exp}|}{|M_U \cup M_{exp}|} \end{aligned}$$

8 $$\begin{aligned} DSC(M_U, M_{exp}) = \frac{2 \cdot |M_U \cap M_{exp}|}{|M_U| + |M_{exp}|} \end{aligned}$$

It is important to note that while *IoU* and *DSC* provide a rigorous independent benchmark, these metrics can be conservative in this specific application. The exact boundaries and geometric shape of the expert-annotated masks are somewhat subjective, as the primary objective is not pixel-perfect edge matching, but rather the reliable identification of a region where respiratory motion is present. Since the predicted masks remain strictly within the torso boundaries (as evidenced by the $S_{geo} \approx 0.99$ with whole torso masks), any significant overlap with the expert ROI ensures that a high-quality, physiologically relevant respiratory signal can be extracted, which is the ultimate functional requirement of the system.

The average $S_{geo}$ of 0.787 indicates high overall alignment, with lower values occurring only in cases where the network-generated shape slightly deviated from the circular ALA template.To verify the anatomical placement, the masks were compared against manual whole-torso masks, yielding an average $S_{geo}$ of 0.987. This confirms that the predicted ROIs are strictly localized within the infant’s torso. Finally, to address the requirement for independent ground-truth verification, we compared the network outputs against expert-annotated R-ROI masks on a held-out test set. The $U{{\text {-}}}Net_1$ achieved an IoU of 0.56 and a Dice score of 0.71, while the DeepLabV3+ model reached an IoU of 0.58 and a Dice score of 0.74.

We also evaluated $U{{\text {-}}}Net_1$ masks against manually annotated full-torso masks ($D_{man}$) to verify that the predicted masks are generally located on the trunk. The sixth row of Table 2 shows that $U{{\text {-}}}Net_1$ trained on $D_{ala}$ achieves a high average $S_{geo}$ with respect to the manually annotated masks. Figure 8 presents typical detection masks.

**Table 2:** Segmentation performance evaluation

U-Net based ROI detector	performance
IoU calculated on U-Net (trained on $D_{ala}$)	0.56
Dice calculated on U-Net (trained on $D_{ala}$)	0.71
IoU calculated on DeepLabV3+ (trained on $D_{ala}$)	0.58
Dice calculated on DeepLabV3+ (trained on $D_{ala}$)	0.74
$S_{geo}>$0.9 with ALA masks	0.935
Avg $S_{geo}$ with ALA masks	0.787
Avg $S_{geo}$ with manual whole torso masks ($D_{man}$)	0.987

Segmentation performance evaluation of the U-Net-based ROI generation ($U{{\text {-}}}Net_1$) where the network was trained on training set from $D_{ala}$. The first two rows are frame based statistics (number of good frames / all frames). The third row shows the average of the particular $S_{geo}$ values. The fourth row shows average $S_{geo}$ value as well, however here the similarity is calculated with the manually generated labels with the entire torso belongs to $D_{man}$

### RR performance analyses

In the second level of evaluation, we assessed the practical performance of ROI detection for respiration rate (RR) estimation using the previously introduced RR estimator [6].

To quantify inter-subject variability and robustness, RR estimation was analyzed separately for each independent hold-out infant across multiple recording segments, including segments with mild motion and caregiving-related disturbances, as summarized in Table 3.

RR estimation performance on independent hold-out subjects is summarized in Table 3. For each infant and recording segment, mean absolute error (MAE), root mean square error (RMSE), 95% confidence intervals (CI), and coverage rates are reported. The 95% CIs were computed using block bootstrap resampling over RR windows.

**Fig. 9:**
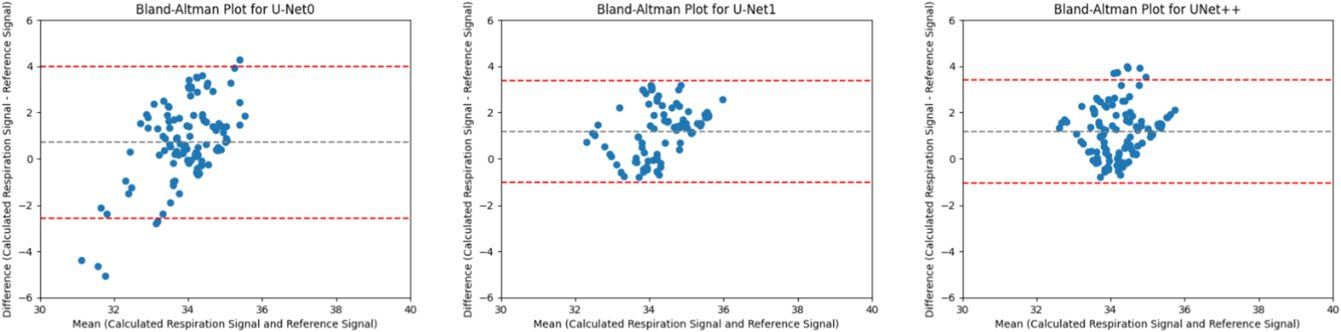
Bland–Altman plots comparing reference respiratory rates and the respiratory rates derived from the RR estimator using different ROIs provided by $U{{\text {-}}}Net_0$, $U{{\text {-}}}Net_1$, and *U-Net++*. The plots were calculated on a representative video segment from an additional independent infant not included in the aggregated RR statistics reported in Table 3. The x-axis represents the average of the two measurements, while the y-axis shows the difference between the measurements. The black dashed line indicates the mean difference (bias), and the red dashed lines denote the limits of agreement

MAE and RMSE were calculated between the ECG-derived ground truth and the estimated respiration rates for each independent hold-out infant (Fig. 9).

**Table 3:** Per-infant respiration rate (RR) estimation performance on independent hold-out subjects.

Infant	MAE (RPM)	RMSE (RPM)	95% CI (MAE)	Coverage Rate	Failure Rate
Infant A (Segment 1)	5.49	8.20	[4.51–6.58]	1.00	0.00
Infant A (Segment 2)	4.89	6.52	[3.74–6.18]	1.00	0.00
Infant C (Segment 3)	2.01	4.10	[1.17–3.10]	1.00	0.00

Respiration rate estimation performance reported separately for each independent hold-out infant and recording segment. The 95% confidence intervals (CI) were estimated using block bootstrap resampling over RR windows. Coverage rate indicates the proportion of valid RR windows relative to the total available windows. Failure rate is defined as $1 - \text {coverage rate}$, representing the proportion of windows where RR estimation was not available

The per-infant results summarized in Table 3 demonstrate stable RR estimation across independent subjects. All neural architectures show improved performance compared to the scenario without ROI detection. The consistently high coverage rates indicate that the proposed approach provides reliable RR estimates across diverse recording conditions, including periods with mild motion and caregiving-related disturbances.

As noted earlier, visual inspection of the generated masks confirmed that all predicted masks were located over regions where respiratory motion was clearly detectable on the torso. In other words, the detector successfully identified physiologically relevant ROIs in each frame.

### Additional architecture validation

To demonstrate that ALA-generated labels are not restricted to U-Net-based architectures, we additionally trained a DeepLabV3+ segmentation network using the same automatically annotated dataset. The training converged reliably without any manual annotations, and the resulting model produced stable and spatially accurate respiration-ROI predictions.

**Fig. 10:**
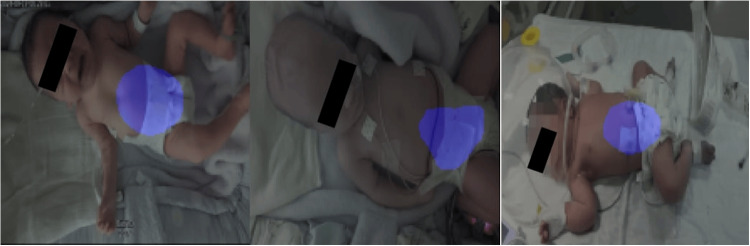
Qualitative results from the DeepLabV3+ model trained exclusively on ALA-generated labels. The predicted respiration regions of interest (ROIs) are shown in blue

Representative qualitative examples are shown in Fig. 10, illustrating robust segmentation performance across different infant poses, lighting conditions, and backgrounds. The dataset includes neonates in open incubators under varying illumination levels, with occasional caregiver movements visible in the background, demonstrating that the algorithm successfully handles diverse visual contexts and can generalize to a range of typical NICU scenarios. These results confirm that the ALA-generated dataset is suitable for training a broad range of modern segmentation architectures, supporting the generality and practical utility of the proposed automatic labeling framework.

## Discussion

### Comparison with state-of-the-art approaches

Among the automatic annotation methods reported in the literature, notable examples include [28] and [29]. Compared to our approach, CycleGAN can be an effective solution for automatic annotation in relatively simple datasets. In these studies, U-Net was trained on the generated datasets to solve specific segmentation tasks. Based on the findings of [28] and [29], CycleGAN and RailGAN are capable of generating datasets containing surface defects or railway errors with a relatively small number of samples. Specifically, in [28], CycleGAN generates an erroneous image from an error-free input, and the difference between the generated and error-free images provides pixel-level segmentation annotations. In the case of RailGAN, the generated erroneous images were labeled using Roboflow.

While generative models like CycleGAN are well-suited for creating such simpler erroneous images and annotations, they are less likely to succeed in handling more complex tasks, such as locating the trunk of infants and generating a dataset for this purpose. Furthermore, they would probably require more annotated reference data for training. In contrast, our method does not rely on reference annotations, as it uses classical algorithms for automatic annotation specifically developed for this application.

The proposed algorithm proved to be effective in generating an annotated dataset without relying on initial reference annotations. The automatically generated annotations were successfully used to train semantic segmentation networks, such as U-Net and DeepLabV3+, enabling reliable detection of the respiratory region of interest (ROI) in images containing infants. The validity of the generated annotations is supported by the fact that the detected regions were consistently located on the infant’s torso (average $S_{geo}$ with manual whole torso masks is 98.7%) and showed satisfying overlap with manually created annotations (see Table 2). Furthermore, the identified regions allowed for stable and physiologically meaningful respiratory signal extraction, confirming the suitability of the generated dataset for respiration-related analysis (see Table 3).

It is also important to note that the proposed Automatic Labeling Algorithm (ALA) was not designed as a general-purpose, real-time respiration detector, but as a practical tool to automatically generate high-quality annotations from typical, periodic respiratory motion. The reliance on periodicity is therefore a deliberate design choice rather than a limitation: ALA performs best under stable breathing conditions, which dominate neonatal recordings, and may simply abstain from labeling in very irregular or motion-corrupted cases. These automatically labeled frames can then be used to train deep learning–based ROI detectors that generalize better to non-periodic or noisy conditions, thus bridging the gap between traditional and learning-based approaches.

Additionally, while the current experiments focused on training U-Net architectures, the ALA-generated dataset can be used to train other segmentation models, such as DeepLabV3+ or SegNet (see Fig. 10). This flexibility underscores the generality of the automatic annotation approach and its potential applicability across a variety of deep learning–based ROI detection frameworks.

### Limitations

A key limitation of the ALA detection component, when used directly as a basis for a training set, is that it may fail to provide valid R-ROI detection in mission-critical situations, such as during apnea. Detection can fail in static scenarios or when other periodic motions are present, such as blinking lights, moving shadows, or movements induced by caregiving or feeding. However, in such cases, ALA simply does not generate a label, and these frames are excluded from training. Consequently, the algorithm can automatically produce an annotated dataset from typical video sequences, which can then be used to train a more robust deep learning–based ROI detector (U-Net). Unlike ALA, this network analyzes static image features and is capable of detecting the R-ROI even if the subject is moving independently of respiration or is completely still.

In addition to algorithmic limitations, the validation dataset itself is relatively small and homogeneous, reflecting the strict ethical and clinical constraints of NICU data collection. All recordings were obtained under real clinical conditions, using the same monitoring setup, where only a single respiratory reference modality (ECG impedance) is routinely available. Although this constrains variability, it also ensures that the dataset authentically represents the typical monitoring environment of preterm infants. Comparable open datasets do not exist, as NICU recordings are rarely shareable due to privacy and consent restrictions.

A limitation of the current study is that the respiratory ground truth was derived from a single modality, namely ECG electrode impedance. While impedance pneumography is the standard of care in the NICU, it is a surrogate measure of thoraco-abdominal motion rather than a direct measure of airflow. Consequently, it may diverge from true ventilation during obstructive events or periods of high motion artifact.

Finally, while the current evaluation focuses on respiration rate estimation accuracy, it does not yet address clinically significant event detection (e.g., apnoea, bradypnoea, tachypnoea). This was beyond the scope of the present work, which primarily aimed to develop and validate the automatic labeling process.

## Future work

We anticipate that this concept can be extended to other applications where the objective is to detect objects in image sequences based on characteristic motion patterns and periodicity. Future work will focus on validating the method in a multicentre setting, incorporating recordings from a larger and more diverse neonatal population. These efforts are already part of our ongoing project roadmap and aim to further strengthen the clinical generalizability of the proposed framework. Additionally, future extensions may involve adapting ALA to operate on nighttime infrared recordings, where color information is unavailable, and exploring approaches for detecting ROIs directly from difference images in cases where infants are partially or fully covered.

## Conclusion

In this work, we introduced the Automatic Labeling Algorithm (ALA), a motion-periodicity–based framework that automatically generates respiration-related region-of-interest (R-ROI) masks from neonatal video recordings. The primary contribution of this study is demonstrating that ALA can reliably produce large volumes of respiration-related annotations without any manual labeling effort.

These automatically generated datasets can then be used to train a wide range of segmentation architectures (including U-Net variants and DeepLabV3+) which learn to detect R-ROIs robustly and independently of periodicity. Our experiments confirm that networks trained purely on ALA-generated labels achieve stable and clinically meaningful ROI localization performance. Overall, ALA effectively addresses the annotation bottleneck in neonatal respiratory monitoring and provides a scalable pathway for training strong deep-learning-based ROI detectors.

Overall, these findings demonstrate that automatic annotation based on motion extent and periodicity, combined with the training of a deep learning–based detector, provides a scalable and effective solution for infant respiration monitoring.

Beyond this specific application, the proposed framework may have the potential to generalize to other domains where periodically moving objects need to be detected in image sequences, and it could offer a promising strategy for automatic dataset generation in challenging environments.

## Data Availability

Restrictions apply to the availability of these data. The dataset was collected under informed consent and is available from the authors upon reasonable request and with appropriate permissions. The full implementation of the proposed Automatic Labeling Algorithm (ALA), along with the segmentation training scripts (including U-Net, U-Net++ and DeepLabV3+), is publicly available. The ALA pipeline is available at: GitHub repository. The segmentation training scripts are available at: DeepLabV3+ repository. The RR evaluation code is available at: RR evaluation repository.

## References

[1] Maurya L, Kaur P, Chawla D, Mahapatra P (2021) Non-contact breathing rate monitoring in newborns: a review. Comput Biol Med 132:104321. 10.1016/j.compbiomed.2021.104321 33773194 10.1016/j.compbiomed.2021.104321

[2] Jorge J, Villarroel M, Chaichulee S, Guazzi A, Davis S, Green G, McCormick K, Tarassenko L (2017) Non-contact monitoring of respiration in the neonatal intensive care unit, pp 286–293. 10.1109/FG.2017.44

[3] Sun Y, Wang W, Long X, Meftah M, Tan T, Shan C, Aarts R, With P (2019) Respiration monitoring for premature neonates in nicu. Appl Sci 9:5246. 10.3390/app9235246

[4] Nagy A, Chetverikov D, Zarándy A (2019) Novel methods for video-based respiration monitoring of newborn babies. In: 2019 In: Képfeldolgozók, és Alakfelismerők Társasága Képfeldolgozók és Alakfelismerők Társaságának 12. Országos Konferenciája, pp 1–10. http://eprints.sztaki.hu/id/eprint/9699

[5] Zarandy A, Foldesy P, Nagy A, Jánoki I, Terbe D, Siket M, Szabo M, Varga J (2020) Multi-level optimization for enabling life critical visual inspections of infants in resource limited environment. In: 2020 IEEE international symposium on circuits and systems (ISCAS), pp. 1–5. 10.1109/ISCAS45731.2020.9181040

[6] Nagy A, Jánoki I, Foldesy P, Terbe D, Siket M, Szabo M, Varga J, Zarandy A (2021) Continuous camera-based premature-infant monitoring algorithms for nicu. Appl Sci 11:7215. 10.3390/app11167215

[7] Katz E, Mitchell R, D’Ambrosio C (2011) Obstructive sleep apnea in infants. Am J Respir Crit Care Med 185:805–16. 10.1164/rccm.201108-1455CI 22135346 10.1164/rccm.201108-1455CI PMC5448577

[8] Ghafoor T, Mahmud S, Ali S, Dogar S (2003) Incidence of respiratory distress syndrome. J College Phys Surgeons-Pakistan JCPSP 13:271–3 12757676

[9] Demeter P, Pap A (2004) The relationship between gastroesophageal reflux disease and obstructive sleep apnea. J Gastroenterol 39:815–20. 10.1007/s00535-004-1416-8 15565398 10.1007/s00535-004-1416-8

[10] Sale S (2010) Neonatal apnoea. Best Pract Res Clin Anaesthesiol 24:323–36. 10.1016/j.bpa.2010.04.002 21033010 10.1016/j.bpa.2010.04.002

[11] Long X, Otte RA, Sanden E, Werth J, Tan T (2019) Video-based actigraphy for monitoring wake and sleep in healthy infants: a laboratory study. MDPI Sensors 19:1075. 10.3390/s19051075 10.3390/s19051075 PMC6432610 30832392

[12] Jorge J, ViIllarroel M, Chaichulee S, McCormick K, Tarassenko L (2018) Data fusion for improved camera-based detection of respiration in neonates. In: Optical diagnostics and sensing XVIII: toward point-of-care diagnostics, p 36. 10.1117/12.2290139

[13] Scebba G, Da Poian G, Karlen W (2020) Multispectral video fusion for non-contact monitoring of respiratory rate and apnea. IEEE Trans Biomed Eng 1–1. 10.1109/TBME.2020.2993649 10.1109/TBME.2020.2993649 32396069

[14] Chaichulee S, Villarroel M, Jorge J, Arteta C, McCormick K, Zisserman A, Tarassenko L (2019) Cardio-respiratory signal extraction from video camera data for continuous non-contact vital sign monitoring using deep learning. Physiol Meas 40. 10.1088/1361-6579/ab525c 10.1088/1361-6579/ab525c PMC7655150 31661680

[15] Pereira C, Yu X, Goos T, Reiss I, Orlikowsky T, Heimann K, Venema B, Blazek V, Leonhardt S, Teichmann D (2018) Noncontact monitoring of respiratory rate in newborn infants using thermal imaging. IEEE Trans Biomed Eng 1–1. 10.1109/tbme.2018.2866878 10.1109/TBME.2018.2866878 30139045

[16] Abbas AK, Heimann K, Jergus K, Orlikowsky T, Leonhardt S (2011) Neonatal non-contact respiratory monitoring based on real-time infrared thermography. Biomed Eng Online 10:93. 10.1186/1475-925X-10-93 22243660 10.1186/1475-925X-10-93 PMC3258209

[17] Villarroel M, Chaichulee S, Jorge J, Davis S, Green G, Arteta C, Zisserman A, McCormick K, Watkinson P, Tarassenko L (2019) Non-contact physiological monitoring of preterm infants in the neonatal intensive care unit. NPJ Digit Med 2:128. 10.1038/s41746-019-0199-5 31872068 10.1038/s41746-019-0199-5 PMC6908711

[18] Rossol S, Yang J, Toney-Noland C, Bergin J, Basavaraju C, Kumar P, Lee H (2020) Non-contact video-based neonatal respiratory monitoring. Children 7:171. 10.3390/children7100171 33036226 10.3390/children7100171 PMC7600716

[19] Foldesy P, Zarandy A, Szabo M (2020) Reference free incremental deep learning model applied for camera-based respiration monitoring. IEEE Sens J 1–1. 10.1109/JSEN.2020.3021337

[20] Kaewtae W, Visitsattapongse S, Pintavirooj C (2024) Detect and estimate the breathing rate of newborn in the infant incubator using contactless sensor. In: 2024 16th Biomedical engineering international conference (BMEiCON). IEEE, pp 1–5

[21] Hashim HA (2024) Non-contact automatic respiratory rate monitoring for newborns using digital camera technology and deep learning. J Biomed Photon Eng 10(4):168–180

[22] Reyes M, Dorta Palmero J, Diaz J, Aragon Perez E, Taboada-Crispi A (2019) Computer vision-based estimation of respiration signals. IFMBE Proc 252–261. 10.1007/978-3-030-30648-9_33

[23] Massaroni C, Presti D, Formica D, Silvestri S, Schena E (2019) Non-contact monitoring of breathing pattern and respiratory rate via rgb signal measurement. Sensors 19:2758. 10.3390/s19122758 31248200 10.3390/s19122758 PMC6631485

[24] Benabdelkader C, Davis L (2002) Detection of people carrying objects: a motion-based recognition approach. In: 2002 5th IEEE international conference on automatic face gesture recognition (FGR), pp 378–383. 10.1109/AFGR.2002.1004183

[25] Al-mutib K, Emaduddin M, Alsulaiman M, Hedjar R, Mattar E (2014) Motion periodicity based pedestrian detection and particle filter based pedestrian tracking using stereo vision camera. Int J Comput Appl Technol 50(1/2):113. 10.1504/ijcat.2014.063913

[26] Zijuan L, Wang L, Liu W, Li B (2016) Human movement detection and gait periodicity analysis using channel state information. In: 2016 12th International conference on mobile ad-hoc and sensor networks (MSN), pp 167–174. 10.1109/MSN.2016.035

[27] Liu Y, Gu X, Huang L, Ouyang J, Liao M, Wu L (2020) Analyzing periodicity and saliency for adult video detection. Multimed Tools Appl 79. 10.1007/s11042-019-7576-6

[28] Tsai D-M, Fan S-K, Chou Y-H (2021) Auto-annotated deep segmentation for surface defect detection. IEEE Trans Instrument Meas 1–1. 10.1109/TIM.2021.3087826

[29] Xia Y, Han S, Kwon H (2023) Image generation and recognition for railway surface defect detection. Sensors 23:4793. 10.3390/s23104793 37430706 10.3390/s23104793 PMC10223381

[30] Reza M, Naik A, Kai C, Crandall D (2019) Automatic annotation for semantic segmentation in indoor scenes. In: 2019 IEEE/RSJ international conference on intelligent robots and systems (IROS). 10.1109/IROS40897.2019.8968230

[31] Ros G, Sellart L, Materzynska J, Vázquez D, López A (2016) The synthia dataset: A large collection of synthetic images for semantic segmentation of urban scenes. In: 2016 IEEE conference on computer vision and pattern recognition (CVPR), pp 3234–3243. 10.1109/CVPR.2016.352

[32] Duchenne O, Laptev I, Sivic J, Bach F, Ponce J (2009) Automatic annotation of human actions in video, pp 1491–1498. 10.1109/ICCV.2009.5459279

[33] Marcu A, Costea D, Licaret V, Leordeanu M (2019) Towards automatic annotation for semantic segmentation in drone videos

[34] Sapkota R, Paudel A, Karkee M (2024) Zero-shot automatic annotation and instance segmentation using llm-generated datasets: eliminating field imaging and manual annotation for deep learning model development. arXiv:2411.11285

[35] Chen R, Lu M, Chen T, Williamson D, Mahmood F (2021) Synthetic data in machine learning for medicine and healthcare. Nat Biomed Eng 5:1–5. 10.1038/s41551-021-00751-8 34131324 10.1038/s41551-021-00751-8 PMC9353344

[36] Tang Q, Chen Z, Ward R, Elgendi M (2020) Synthetic photoplethysmogram generation using two gaussian functions. Sci Rep 10:13883. 10.1038/s41598-020-69076-x 32807897 10.1038/s41598-020-69076-x PMC7431427

[37] Shin H-C, Orton M, Collins D, Doran S, Leach M (2011) Autoencoder in time-series analysis for unsupervised tissues characterisation in a large unlabelled medical image dataset. In: 2011 10th International conference on machine learning and applications (ICMLA), vol 1, p 259. 10.1109/ICMLA.2011.38

[38] Tang Q, Chen Z, Allen J, Alian A, Menin C, Ward R, Elgendi M (2020) Ppgsynth: an innovative toolbox for synthesizing regular and irregular photoplethysmography waveforms. Front Med. 10.3389/fmed.2020.597774 33224967 10.3389/fmed.2020.597774 PMC7668389

[39] Zhu F, Fei Y, Fu Y, Liu Q, Shen B (2019) Electrocardiogram generation with a bidirectional lstm-cnn generative adversarial network. Sci Rep 9. 10.1038/s41598-019-42516-z 10.1038/s41598-019-42516-z PMC6494992 31043666

[40] Tokmakov P, Alahari K, Schmid C (2017) Learning video object segmentation with visual memory. In: 2017 IEEE international conference on computer vision (ICCV), pp 4491–4500. 10.1109/ICCV.2017.480

[41] Hur J, Roth S (2020) Optical flow estimation in the deep learning age, pp 119–140. 10.1007/978-3-030-46732-6_7

[42] Weinzaepfel P, Revaud J, Harchaoui Z, Schmid C (2013) Deepflow: Large displacement optical flow with deep matching. In: 2013 IEEE international conference on computer vision, pp 1385–1392. 10.1109/ICCV.2013.175

[43] Haan G, Biezen P, Huijgen H, Ojo O (1993) True motion estimation with 3-d recursive search block matching. Circ Syst Vid Technol IEEE Trans 3:368–379388. 10.1109/76.246088

[44] Bolelli F, Allegretti S, Baraldi L, Grana C (2019) Spaghetti labeling: Directed acyclic graphs for block-based connected components labeling. IEEE Trans Image Process 1–1. 10.1109/TIP.2019.2946979 10.1109/TIP.2019.2946979 31634837

[45] Jolliffe IT (2002) Principal Component Analysis. Springer, New York. 10.1007/b98835

[46] Fleming S, Thompson M, Stevens R, Heneghan C, Plüddemann A, Maconochie I, Tarassenko L, Mant D (2011) Normal ranges of heart rate and respiratory rate in children from birth to 18 years of age: a systematic review of observational studies. Lancet 377:1011–8. 10.1016/S0140-6736(10)62226-X 21411136 10.1016/S0140-6736(10)62226-X PMC3789232

[47] Horn B, Schunck B (1981) Determining optical flow. Artif Intell 17(1–3):185–203. 10.1016/0004-3702(81)90024-2

[48] Lucas B, Kanade T (1981) An iterative image registration technique with an application to stereo vision (ijcai), vol 81

[49] Ronneberger O, Fischer P, Brox T (2015) U-net: convolutional networks for biomedical image segmentation. LNCS 9351:234–241. 10.1007/978-3-319-24574-4_28

[50] Zhou Z, Rahman Siddiquee MM, Tajbakhsh N, Liang J (2019) Unet++: redesigning skip connections to exploit multiscale features in image segmentation. IEEE Trans Med Imaging. 10.1109/TMI.2019.2959609 31841402 10.1109/TMI.2019.2959609 PMC7357299

[51] Farnebäck G (2003) Two-frame motion estimation based on polynomial expansion. Image Anal 2749:363–370. 10.1007/3-540-45103-X_50

[52] Nagy A (2022) Neonatal-respiration-monitoring-algorithm. GitHub

[53] GitHub - wkentaro/labelme: Image Polygonal Annotation with Python (polygon, rectangle, circle, line, point and image-level flag annotation).–github.com. https://github.com/wkentaro/labelme. Accessed 22 Jan 2026

